# Comparative study between laser and conventional techniques for class V cavity preparation in gamma-irradiated teeth (*in vitro* study)

**DOI:** 10.1590/1678-7757-2016-0663

**Published:** 2017

**Authors:** Amr H. M. Rasmy, Tarek A. Harhash, Rami M. S. Ghali, Eman M. F. El Maghraby, Dalia H. El Rouby

**Affiliations:** 1 Egyptian Atomic Energy Authority, National Centre for Radiation Research and Technology (NCRRT), Health Radiation Research Department, Cairo, Egypt; 2 Cairo University, National Institution of Laser Enhanced Sciences, Medical Laser Applications Department, Cairo, Egypt; 3 Aachen University, Aachen Dental Laser Center, Aachen, Germany; Ain Shams University, Cairo, Egypt; 4 Cairo University, Faculty of Oral & Dental Medicine, Oral Pathology Department, Cairo, Egypt

**Keywords:** Laser, Gamma ray

## Abstract

**Objective:**

The purpose of this study was to compare laser with conventional techniques in class V cavity preparation in gamma-irradiated teeth.

**Methods:**

Forty extracted human teeth with no carious lesions were used for this study and were divided into two main groups: Group I (n = 20) was not subjected to gamma radiation (control) and Group II (n=20) was subjected to gamma radiation of 60 Gray. Standard class V preparation was performed in buccal and lingual sides of each tooth in both groups. Buccal surfaces were prepared by the Er,Cr:YSGG laser (Waterlase iPlus) 2780 nm, using the gold handpiece with MZ10 Tip in non-contact and the “H” mode, following parameters of cavity preparation – power 6 W, frequency 50 Hz, 90% water and 70% air, then shifting to surface treatment laser parameters – power 4.5 W, frequency 50 Hz, 80% water and 50% air. Lingual surfaces were prepared by the conventional high-speed turbine using round diamond bur. Teeth were then sectioned mesio-distally, resulting in 80 specimens: 40 of which were buccal laser-treated (20 control and 20 gamma-irradiated specimens) and 40 were lingual conventional high-speed bur specimens (20 control and 20 gamma-irradiated specimens).

**Results:**

Microleakage analysis revealed higher scores in both gamma groups compared with control groups. Chi-square test revealed no significant difference between both control groups and gamma groups (p=1, 0.819, respectively). A significant difference was revealed between all 4 groups (p=0.00018).

**Conclusion:**

Both laser and conventional high-speed turbine bur show good bond strength in control (non-gamma) group, while microleakage is evident in gamma group, indicating that gamma radiation had a dramatic negative effect on the bond strength in both laser and bur-treated teeth.

## Introduction

Radiotherapy is a common therapeutic modality for malignancies of head and neck. It is usually associated with some possible complications such as radiation caries, xerostomia, osteonecrosis, loss of taste and trismus^17^.

The symptoms of microleakage range from postoperative hypersensitivity or loss of the restoration due to bond failure to damage to vital dentin and pulp tissue, which in some cases may be irreversible. Effects of microleakage include marginal discoloration and secondary caries, which are due to the presence of bacteria, their nutrients or hydrogen ions, originating from plaque on the surface and leaking into the interfacial space. Bacterial marginal leakage has been implicated as an etiological factor in recurrent caries and pulp irritation following the application of restorations^9^.

Many different techniques may be used to evaluate microleakage, including air pressure, bacterial studies, radioisotope studies, neutron activation studies, scanning electron microscope, chemical tracers and dye penetration studies^21^ ^,^ ^30^ ^,^ ^31^.

A study performed to investigate the influence of therapeutic dose X-rays on the microhardness and degree of conversion of two different aesthetic restorative dental materials found that the therapeutic dose applied to cured material can promote linking and breaking of chain bonds. Thus, the authors recommended that the confection of a new dental restoration with a photo-cured composite resin should be made after the end of radiotherapy and never before, and old restorations should be attended and replaced when necessary^7^.

Bulucu, et al.^5^ (2009) evaluated the effect of radiotherapy on the microleakage of three adhesive systems and observed no significant difference when comparing different adhesive systems. However, significant differences were observed between enamel and dentin (p<0.01), in which the microleakage at the dentin margins was greater than at the enamel margins.

Naves, et al.^16^ (2012) evaluated the effect of gamma radiation on the microtensile bond strength of resin-based composite restoration to human enamel and dentin performed either before or after radiotherapy. The authors concluded that gamma radiation had a significant detrimental effect on bond strength to human enamel and dentin when the adhesive restorative procedure was carried out after radiotherapy.

Since previous studies evaluated either the effect of laser cavity preparation or gamma irradiation on surface roughness and microleakage, the combined effect of both variables still needs to be investigated.

Therefore, this study was carried out to detect the surface morphology and presence of microleakage in composite resin restoration following etched bur cavity preparation and laser cavity preparation in irradiated teeth.

## Material and methods

In this study, we used 40 molar teeth with no carious lesions, extracted from males of age 40-50 years from the Surgery Department of the Faculty of Dentistry, Cairo University, due to periodontal diseases or in preparation to receive a full denture. Teeth were stored in a 0.1% thymol solution until the study was carried out^26^.

They were divided into two main groups:

Group I (n = 20) was not subjected to gamma radiation (control).
Group II (n = 20) was subjected to gamma radiation of 60 Gray, which is the therapeutic dose for head and neck lesions^11^.

### Radiation exposure

Irradiation of teeth was performed at the National Centre for Radiation Research and Technology (NCRRT), in Cairo, Egypt, using the 137-Cesium source Gammacell^®^ 40, at a dose rate of 0.761 Gy/min at the time of the study.

### Laser application

Buccal surfaces of teeth were prepared by the Er,Cr:YSGG laser (Waterlase iplus) of wavelength 2780 nm (“H” mode), using the gold handpiece with MZ10 tip in non-contact. We used the following parameters during cavity preparation: power 6 W, frequency 50 Hz, 90% water and 70% air, shifting to surface treatment laser parameters of power 4.5 W, frequency 50 Hz, 80% water and 50% air.

### Cavity preparation

A standard class V preparation was performed on buccal and lingual sides of each tooth in both groups with the following dimensions: diameter 3 mm; depth 3 mm; about 2 mm occlusally to the cementenamel junction^25^. Lingual surfaces were prepared by the conventional high-speed turbine using round number 3411 diamond bur, while buccal surfaces were prepared by the Er,Cr:YSGG laser (Waterlase iplus).

Afterwards, teeth were sectioned mesio-distally, resulting in 80 specimens, 40 of which were buccal laser-treated (20 control and 20 gamma-irradiated specimens) and 40 were lingual conventional high­ speed bur specimens (20 control and 20 gamma-irradiated specimens), resulting in the four groups of this study:

Control, laser-treated group (CL);
Control, conventional high-speed bur-treated group (CB);
Gamma, laser-treated group (GL);
Gamma, conventional high-speed bur-treated group (GB).

From each group 10 specimens were subjected to scanning electron microscope (SEM) to study their surface morphology and 10 specimens were used to measure the microleakage, using stereomicroscope.

### Scanning electron microscope (SEM)

To study surface morphology using scanning electron microscopy, specimens were prepared as follows:

Each specimen was dehydrated in graded alcohol (ethanol) series (50, 70, 85, 90 and 100%) for 10 minutes at each concentration, and then coated with a thin film of gold by sputtering coater. Analysis was performed using scanning electron microscopy (SEM, Philips XL30, %600MD, Eindhoven, Netherlands).

### The microleakage test

For measuring the microleakage using stereomicroscope (SZ-DC Olympus, Camera Olympus DC10, Japan), specimens (both control and gamma-irradiated) were prepared as follows:

Each conventional high-speed turbine bur specimen was acid-etched using phosphoric acid 30% gel for 30 seconds and then water sprayed for another 30 seconds and dried with air for 20 seconds, while the laser-treated specimen was used directly without acid etching. Both groups – laser treated and conventional high-speed turbine bur – were subjected to a bonding agent (Adper Single Bond, 3M, ESPE, St. Paul, USA) and adhesive composite (Filtek Z 250, 3M, ESPE, St. Paul, USA, Shade A3) was light-activated using light-curing device (with 800 mw/cm^2^ intensity, Woodpecker, USA) for 20 seconds, according to the manufacturer's instructions. Restorations were kept at room temperature for 24 hours and were finished and polished using rubber cups. The microleakage test was carried out with the whole tooth surface – except for the filled cavities and the region around 1 mm beyond the margins of the cavities – was coated with a nail varnish; then, the specimens were immersed in a 2% methylene blue solution under a thermo-cycling bath for 48 hours, then washed. Specimens were bisected at a bucco-lingual (palatal) plane with a diamond disc (D&Z, Germany), and scored for any microleakage using a stereomicroscope (SZ-DC Olympus, Camera Olympus DC10, Japan)^2^ ^,^ ^25^ ^,^ ^29^.

**Figure 1:**
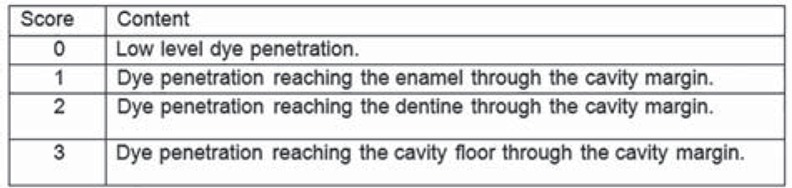
Four grade scale showing degree of micro leakage

The degree of microleakage was assessed using a 4-grade scale (Figure 1) and the technician was not informed of the nature and purpose of the experiment. When both sides of the same specimen revealed different scores, the highest score was recorded.

Statistical analysis was then performed using a commercially available software program (SPSS 19; SPSS, Chicago, IL, USA) to compare the microleakage scores recorded for different groups using the Chi-square test. The level of significance was set at p<0.05.

## Results

### Microleakage analysis

**Figure 2:**
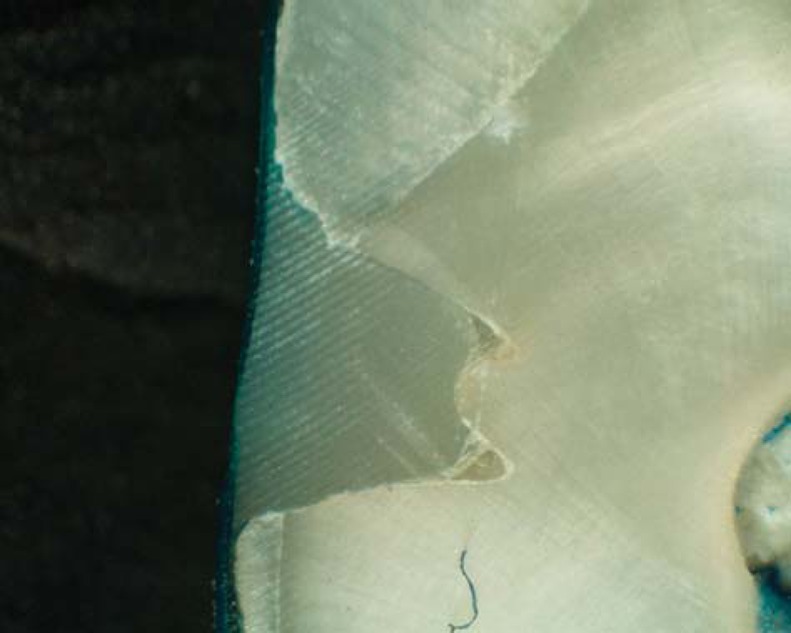
Photomicrograph of control group (laser) showing no micro leakage (score 0)

**Figure 3:**
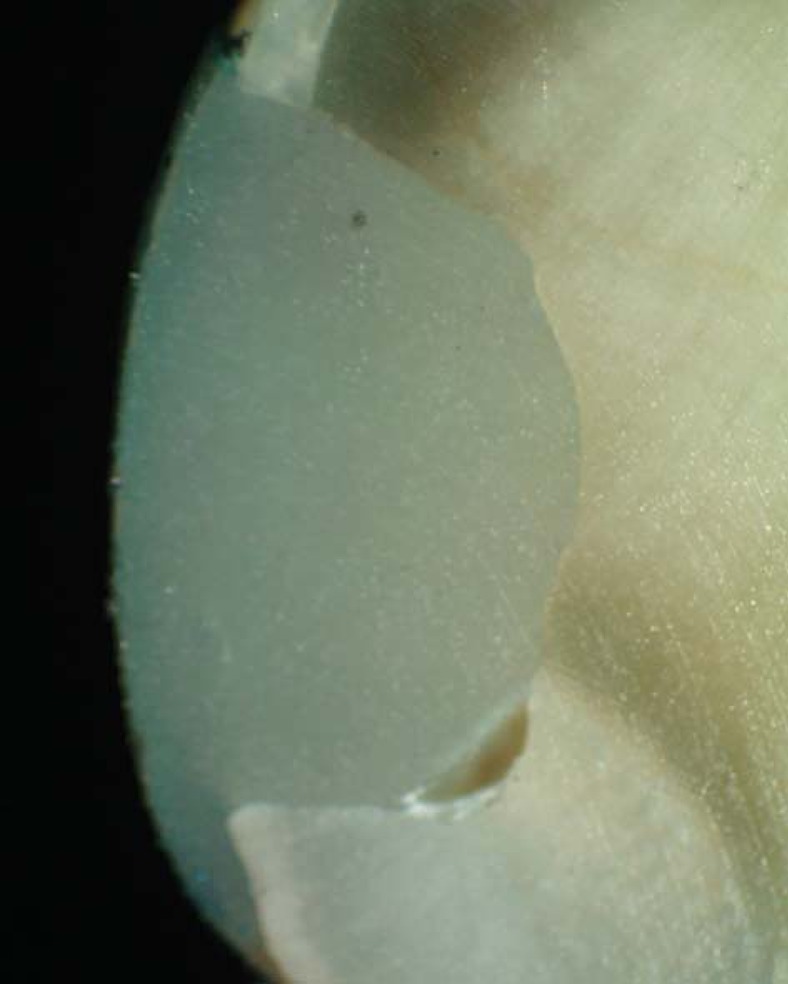
Photomicrograph of control group (bur) showing no micro leakage (score 0)

**Figure 4:**
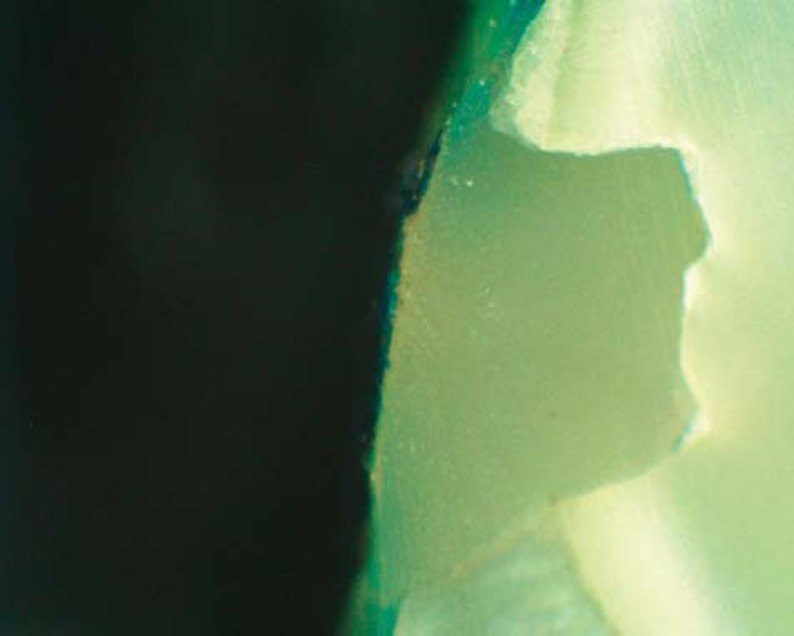
Photomicrograph of gamma group (laser) showing micro leakage (score 3)

**Figure 5:**
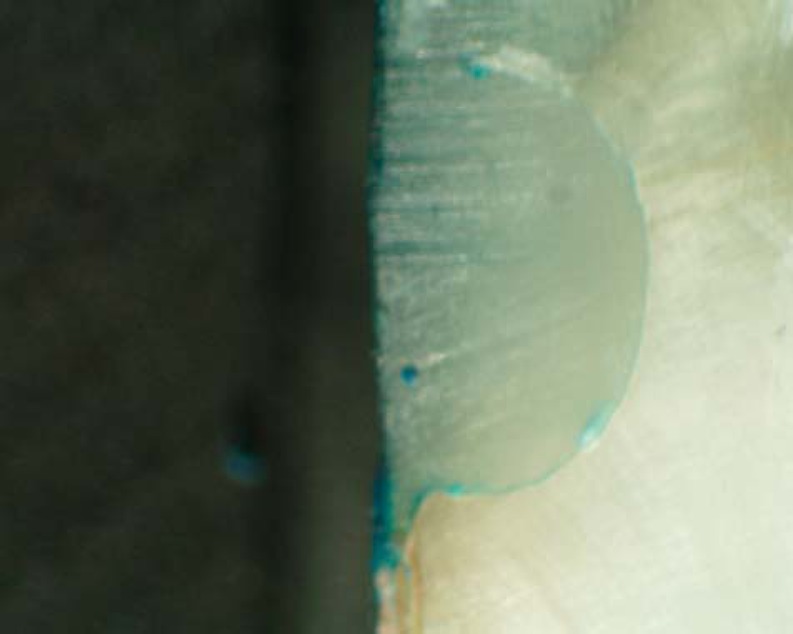
Photomicrograph of gamma group (bur) showing micro leakage (score 3)

**Figure 6:**
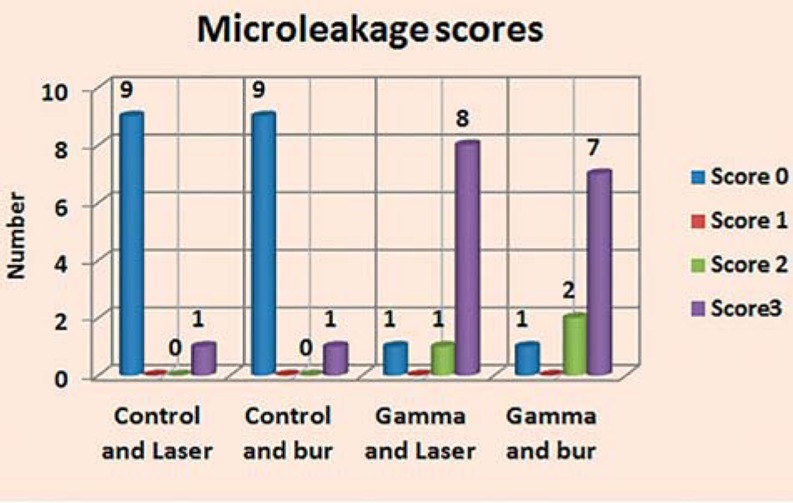
Micro-leakage scores in all groups

Results of microleakage analysis revealed higher scores in both gamma and control groups. The Chi-square test revealed no significant difference between both control groups, neither between both gamma groups (p = 1, 0.819, respectively). A significant difference was revealed in all four groups (p=0.00018) (Table 1, Figures 2-6).

**Table 1:** Microleakage score in all groups and significance of the difference (Chi-square test)

Groups		Score 0	Score 1	Score 2	Score 3	P1 value	P2 value
Control groups	Laser (N=10)	(90%)	0	0	1(10%)	1ns	
	Bur (N=10)	9 (90%)	0	0	1(10%)		
							0.00018*
Gamma groups	Laser (N=10)	1 (10%)	0	1(10%)	8 (80%)	0.819ns	
	Bur (N=10)	1 (10%)	0	2 (20%)	7 (70%)		

ns=non-significant,

* significant at p<0.05

P1: significance of difference between both control and gamma groups

P2: significance of difference between all groups

**Figure 7:**
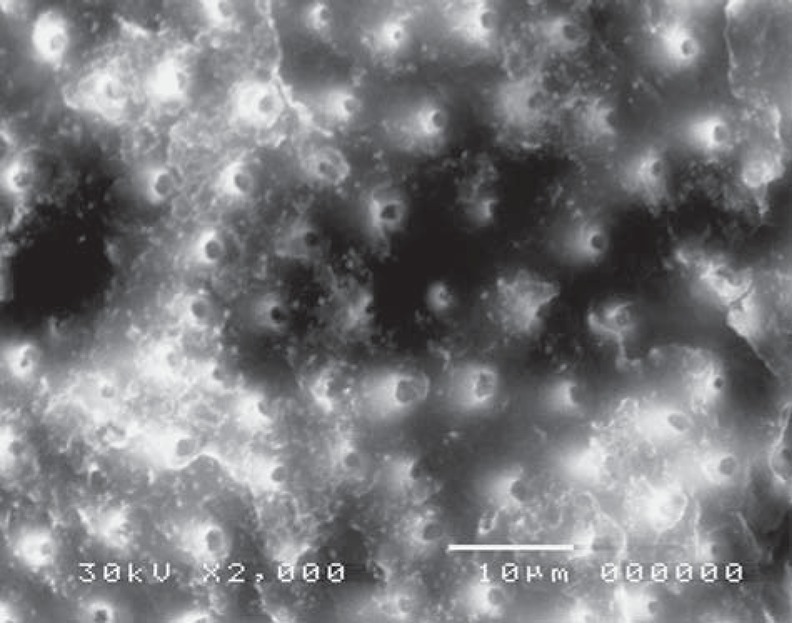
Electron-micrograph in control (CL) group after cavity preparation with laser revealing a rough irregular surface. The smear layer was removed and the surface structure is maintained (×2,000)

**Figure 8:**
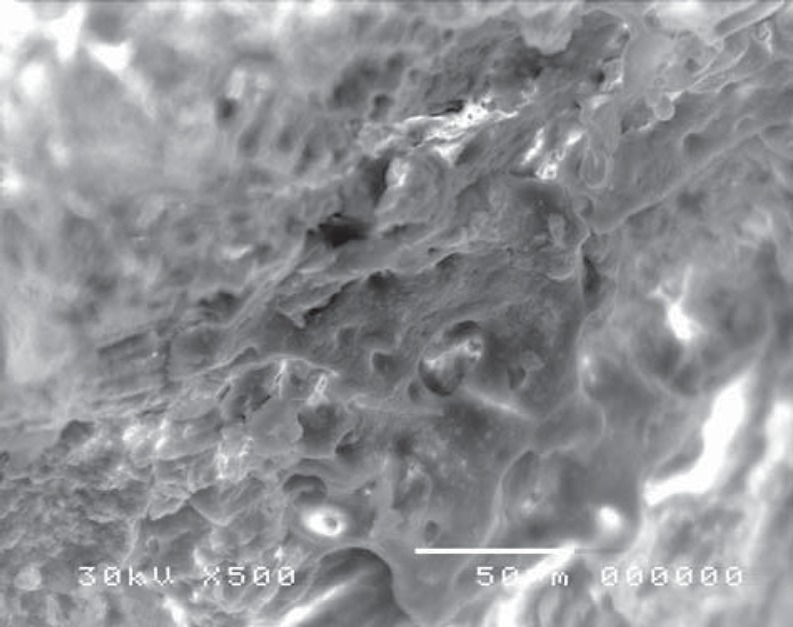
Electron-micrograph in control group (CL) after cavity preparation with laser showing confluence of tubular and intertubular structures in some areas. Irregular inter-lacing collagen fibers, and partially obliterated dentinal tubules are also noted (×500)

**Figure 9:**
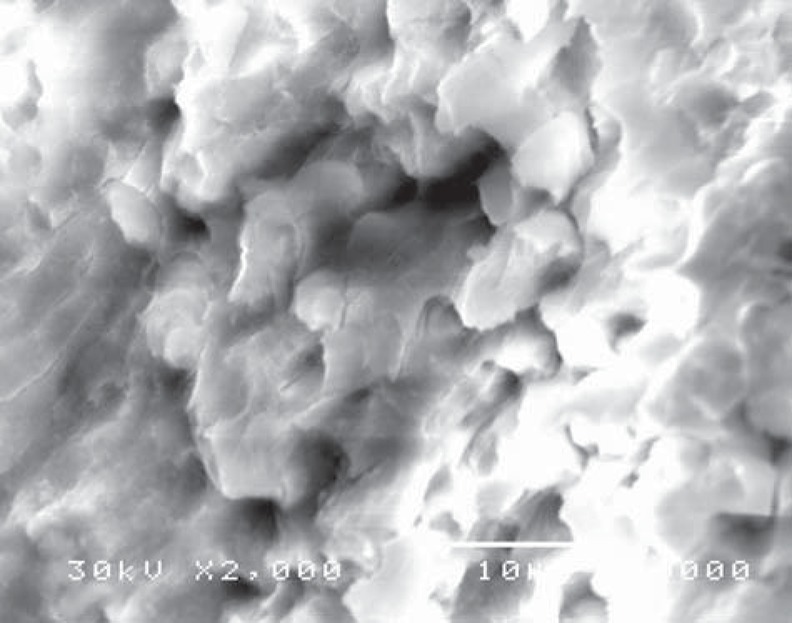
Electron-micrograph in control (CL) group after cavity preparation with laser showing irregular dentinal tubules with partially obliterated lumen intermingled with areas where the tubular structure can't be identified (×2,000)

**Figure 10:**
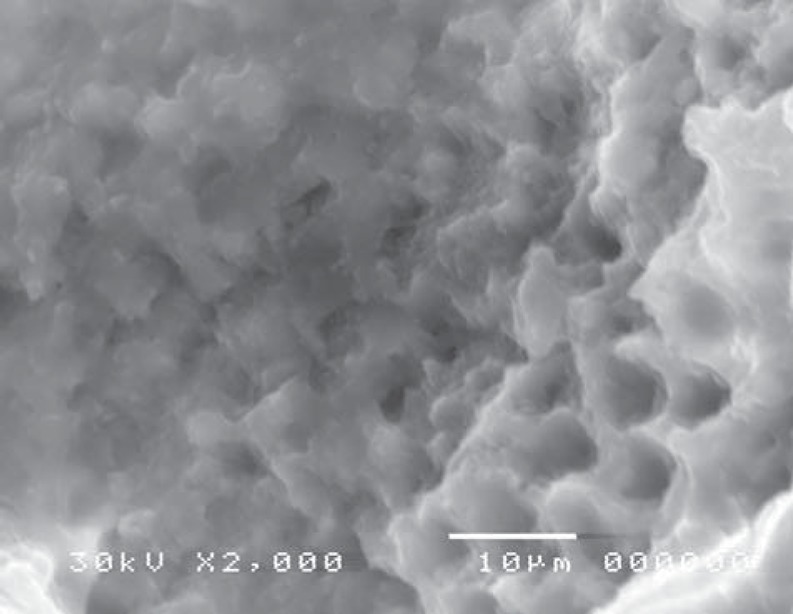
Electron-micrograph in gamma (GL) group subjected to laser showing confluence of tubular and intertubular structures, the intertubular dentine was ablated more than the peritubular dentine, giving the appearance of irregularity and protrusion of the dentinal tubules (×2,000)

Scanning electron microscope revealed structural changes of the dentine surface morphology subjected to laser in the control group (CL). The tubular structure could be identified in some specimens (Figure 7) while in others, the dentine surface revealed confluence of tubular and inter-tubular structures in some areas. Irregular, interlacing collagen fibers and partially obliterated dentinal tubules were also observed (Figures 8, 9). In the gamma laser (GL) group, the inter-tubular dentine was ablated more than the peritubular dentine, giving the appearance of irregularity and protrusion of the dentinal tubules (Figure 10).

**Figure 11:**
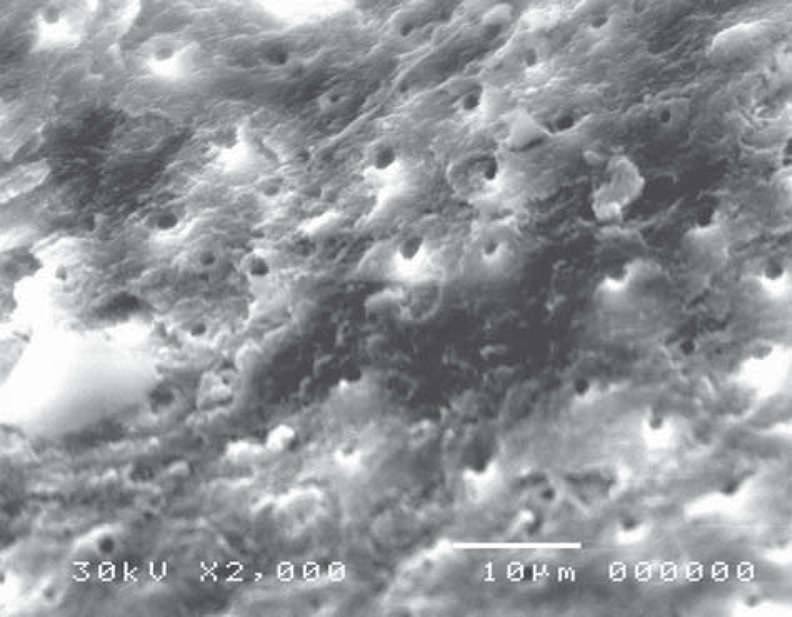
Electron-micrograph in gamma and laser (GL) group showing irregular dentine surface after cavity preparation. The tubular structure is maintained in most areas and reveals variation in tubular diameter (×2,000)

**Figure 12:**
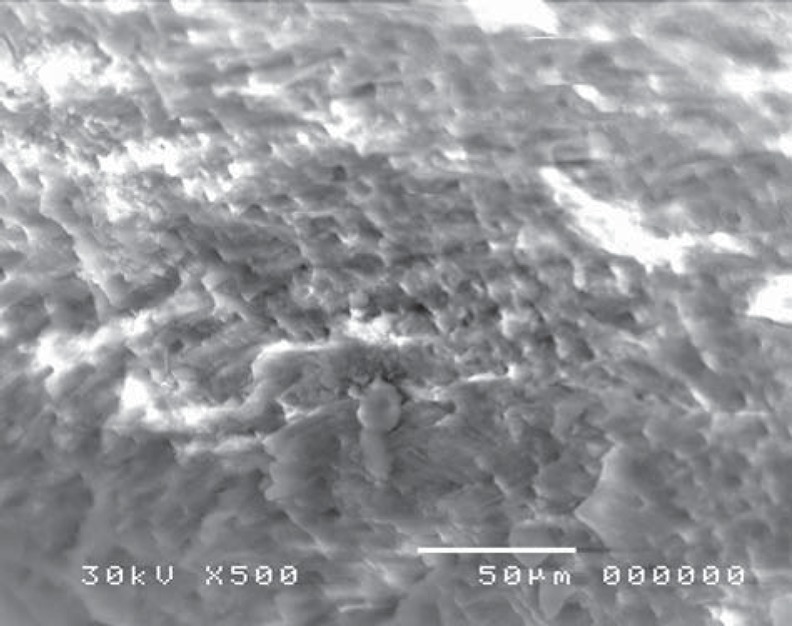
Electron-micrograph in gamma (GL) group subjected to laser showing dentine surface after cavity preparation. Confluence of tubular and inter-tubular structures is noted in some areas, while in other areas, the tubular structure is maintained and reveals variation in tubular diameter (blue arrow), (×500)

Confluence of tubular and inter-tubular structures was noted in some areas, while in others the tubular structure was maintained and revealed variation in tubular diameter with irregular dentine surface (Figures 11, 12).

**Figure 13:**
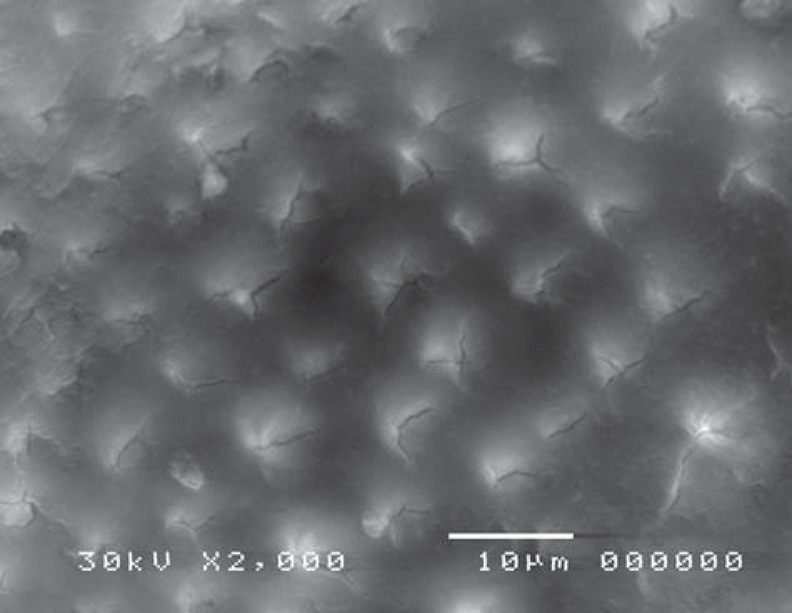
Electron-micrograph in control (CB) group subjected to conventional high speed turbine bur before acid etching showing the dentinal tubules orifices were relatively closed (×2,000)

**Figure 14:**
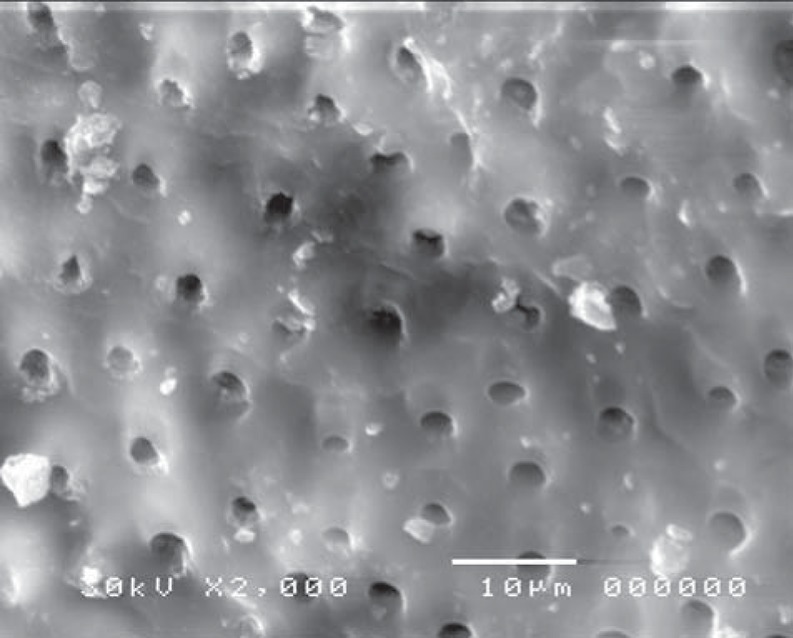
Electron-micrograph in control (CB) group subjected to conventional high speed turbine bur after acid etching where the smear layer was removed and the dentinal tubules were obvious. The intertubular dentine appears more ablated than the peritubular one (×2,000)

On the other hand, in the cavity surface prepared with the conventional high-speed turbine bur in the control (CB) group, dentinal tubules orifices were relatively closed (Figure 13); however, after treatment with 37% phosphoric acid, the smear layer was removed and the dentinal tubules became obvious. The inter-tubular dentine was more ablated than the peritubular one (Figure 14).

**Figure 15:**
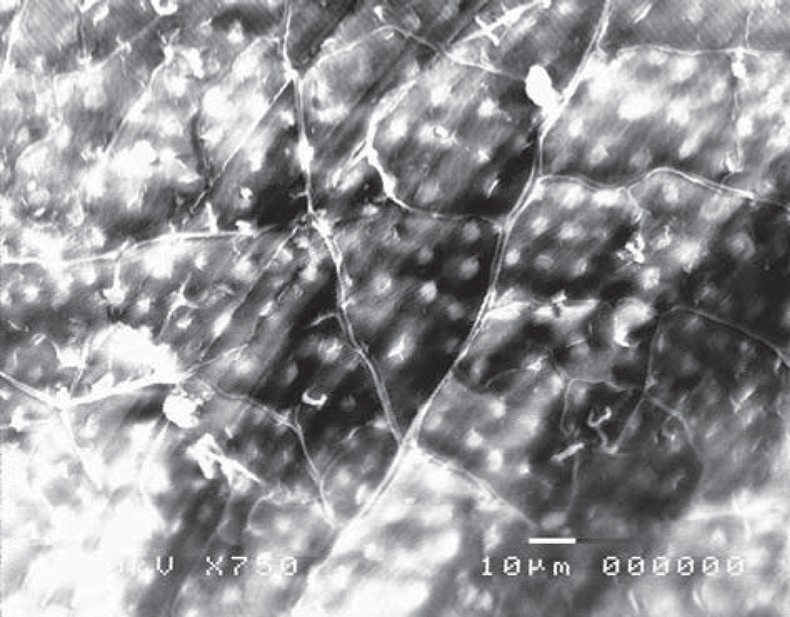
Electron-micrograph in gamma (GB) group subjected to conventional high speed turbine bur after cavity preparation revealing cracks and debris on the dentine surface (×750)

**Figure 16:**
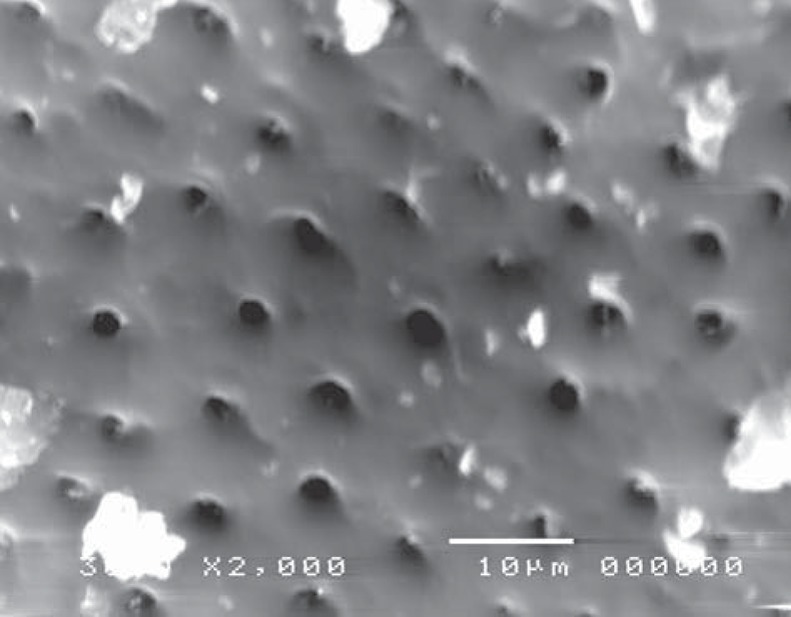
Electron-micrograph in gamma (GB) group subjected to conventional high speed turbine bur after acid etching, the dentinal tubules became more obvious, and the intertubular dentine appears more ablated than the peritubular one and the smear layer was removed (×2,000)

Moreover, specimens prepared with the conventional high-speed turbine bur in gamma (GB) group (before acid etching) revealed a flat surface covered with a smear layer, with many cracks and debris (Figure 15). Similarly to the control group, after treatment with 37% phosphoric acid, the smear layer was removed and the dentinal tubules became obvious, where the inter-tubular dentine appeared more ablated than the peritubular one (Figure 16).

## Discussion

Cavity preparation performed by the Er,Cr:YSSG laser of wavelength 2780 nm showed the possibility to perform cavities within a few minutes. However, the required time for use of the laser was much longer than the use of the conventional high-speed bur because the removal of enamel tissues was more difficult with the use of laser. Hence, cutting through the enamel by laser had a lower efficiency than cutting through the dentine because of the less quantity of water and organic contents of enamel structures if compared with dentine structures^1^.

Laser parameters used in this study revealed that the surface mean temperature did not exceed 4°C that is believed to be safe for pulp vitality^13^.

After performing the microleakage test, some dye may have penetrated into the resin in a muchdiffused way such that score 0 means low-level dye penetration.

Previous morphological studies of enamel surface showed protruding prism sheaths without erosion, while the dentine surface showed exposed dentinal tubules orifices. This is believed to be due to microexplosion effects caused by the hard tissue ablation with the Er,Cr:YSSG laser of wavelength 2780 nm. Moreover, surfaces show scaly or flaky irregular appearance^1^. However, in case of acid etching during normal restorative procedures, chemical changes may lead to increasing dentine permeability and wetness and increasing in pulp irritation, with modification of the organic component and decalcification of the inorganic part of the hard tooth structures^4^ ^,^ ^19^.

According to a study conducted by Yamada, et al.^28^ (2000), who used different dye type and concentration but the same soaking time for measuring the microleakage test, surface roughness influence the microleakage test or bond strength because a rougher surface for a given cross sectional area would have a greater surface area, decreasesing microleakage and increasing bond strength. Therefore, the highly irregular surfaces or roughness without a smear layer in laser cavities could provide a suitable surface for good adhesion or strong bonding with restorative materials in comparison with acid-etched surfaces – thus, it is possible to avoid acid etching^12^ ^,^ ^27^. However, our findings are in agreement with that of Subramaniam and Pandey^26^ (2016), who used the same dye type and concentration but different soaking time for measuring the microleakage test, finding no significant difference in microleakage between composite resin bonded to lased enamel and dentine and teeth prepared with conventional method. The authors also noted that the microleakage observed in both groups was due to the presence of gaps at the resin-tooth interface and that this, in turn, may be due to poor adaptation or less penetration of resin material into dentinal tubules, entrapped air and inadequate curing of the material. Thus, resins with good flow characteristics and surface treatment of laser-prepared cavities have been suggested for better wetting and penetration^29^.

Our findings are in contrast with that of studies of Palma-Dibb, et al.^18^ (2002), Corona, et al.^6^ (2003) and Roebuck, et al.^23^ (2000), who used different dye type, concentration and soaking time for measuring the microleakage test, reporting a higher degree of microleakage around composite restorations when cavity preparation was performed by the Er:YAG laser. Palma-Dibb, et al.^18^ (2002) demonstrated that, by scanning electron microscope (SEM), the morphology of lased surfaces reveals an irregular ablation pattern and non-sufficient etching with presence of unconditioned dental surface areas, which may be due to difficulty in obtaining a uniform pulse administration.

Consequently, laser creates non-uniform microporosities and promotes a disorganized destruction of enamel prisms. These irregular microretentions vary from the acid etching pattern resulting in poor adhesion with adverse effect on effectiveness of cavity margin sealing^19^. Moreover, Corona, et al.^6^ (2003) demonstrated the same results, but attributed them to the cavosurface margins produced by Er:YAG laser irradiation, which provide a quite rough appearance compared to margins produced by high­ speed cutting, thus marginal contouring could result in increased micro-spacing and greater leakage.

Roebuck, et al.^23^ (2000) also stated that the morphological changes on tooth structure caused by laser irradiation might affect the degree of performance of restorative materials, especially adhesives systems. Additionally, increasing the pulse energy might result in deeper crater pattern in tooth surface, which may influence the adaptation of restorative material to cavity walls; therefore, different pulse energies are required for optimum cavity sealing at enamel and dentine margins and for different materials.

The bonding process could be impaired due to the presence of free radicals within the structure of dental tissues previously exposed to ionizing radiation^15^. These free radicals act in a similar way to hydrogen peroxide (O^-^ highly reactive radicals interfere with polymerization)^3^ ^,^ ^20^, sodium hypochlorite (free radicals act on collagen denaturation)^8^, or blood contamination (hemoglobin iron-dependent radicals)^22^.

Moreover, the hydroxylapatite crystals of dental hard tissues contain some sodium, magnesium and carbonate by entrapment during their formation^16^, in which sodium and magnesium may substitute calcium and carbonate can substitute phosphate and hydroxyl group; those substitutions distort the dental structure and make it more soluble^24^. After irradiation, these defects could be mobilized from the surface layer of crystals, removing entrapped ions and modifying the dental crystal structure, thus interfering with adhesion to restoration. This occurs more in enamel, which contains higher inorganic matter compared with dentine^14^ ^,^ ^16^ ^,^ ^24^ ^,^ ^25^. The morphologic, metabolic and compositional alteration in intra- and inter-tubular collagen might have an effect on bond strength to dentin^10^ ^,^ ^16^.

Finally, the high degree of microleakage in gamma group denotes that gamma radiation had a dramatic negative effect on bond strength in both laser and bur-treated teeth. This might be attributed to alteration in the crystalline structure and the chemical composition of both enamel and dentine surface.

## Conclusion

Both laser and conventional high-speed turbine bur may have good bond strength in control (non-gamma) group. However, both groups showed a high degree of microleakage in the gamma group, suggesting that gamma radiation had a dramatic negative effect on bond strength in both laser and bur-treated teeth.
